# Incidence of severe hypoglycaemic episodes in patients with type 2 diabetes in the Basque country: impact on healthcare costs

**DOI:** 10.1186/s12913-015-0876-2

**Published:** 2015-05-27

**Authors:** Edurne Alonso-Morán, Juan F. Orueta, Roberto Nuño-Solinís

**Affiliations:** O+berri, Basque Institute for Healthcare Innovation, Torre del BEC (Bilbao Exhibition Centre), Ronda de Azkue 1, 48902 Barakaldo, Spain; Osakidetza, Basque Health Service, Centro de Salud de Astrabudua, Mezo 35, 48950 Erandio, Spain; Deusto Business School, University of Deusto, Hermanos Aguirre 2, 48014 Bilbao, Spain

**Keywords:** Hypoglycaemia, Type 2 diabetes mellitus, Healthcare costs, Glycosylated haemoglobin, Socioeconomic level

## Abstract

**Background:**

Hypoglycaemia is an acute complication of diabetes mellitus which poses a serious threat. This study aims to describe the annual rate of people suffering episodes of severe hypoglycaemia and to estimate the healthcare costs for individuals who have suffered such events.

**Methods:**

A descriptive study involving all patients with type 2 diabetes (T2DM) from the Basque Country (period: 1/09/2010 to 31/08/2011) aged ≥35 years (N = 134,413). The rate of hypoglycaemic episodes treated in hospitals (Accident and Emergency and in-patient services) was calculated using an algorithm based on diagnostics and laboratory tests. The variables recorded included demographic, comorbidity (diagnoses categorised using the Adjusted Clinical Groups case-mix system) and socioeconomic variables (deprivation index of the area of residence). The annual healthcare cost for people with T2DM who suffered those episodes was compared with those who did not by regression analysis.

**Results:**

The incidence of hypoglycaemia in the Basque Country was 0.56 %. This percentage was higher among women and people with a lower socioeconomic status. These episodes were associated with age and high values of glycosylated haemoglobin (HbA_1c_) > 7 %. Adjusting for the other variables, on average, people who suffered hypoglycaemia accounted for an additional €2509 in annual healthcare costs.

**Conclusions:**

Hypoglycaemia has high morbi-mortality and a major economic impact. As such, health services must monitor its appearance and promote specific actions, especially in the higher risk sub-populations.

## Background

Diabetes mellitus (DM) has become one of the most important challenges faced by healthcare systems due to its high worldwide prevalence and the high economic burden resulting from it. By 2035, it is estimated that around 592 million people will suffer from diabetes, 210 million more than in 2013. In addition, resource use by diabetes patients represented 10.8 % of total world healthcare costs in 2013, ranging between 5 % and 18 % in numerous countries [1].

Although the majority of research into the complications of DM is aimed at studying the impact of chronic complications on the quality of life of diabetes sufferers and the resulting healthcare costs, hypoglycaemic episodes are a common acute complication into this group.

It is widely accepted that hypoglycaemia is an important problem as regards caring for DM patients receiving insulin treatment [2]. However, its importance amongst patients receiving oral antidiabetics may not be as well recognised and the estimated burden resulting from hypoglycaemic episodes in diabetes mellitus type 2 (T2DM) may therefore be underestimated.

Hypoglycaemic episodes may result in severe morbidity, causing vascular events such as stroke, acute myocardial infarction, acute heart failure and ventricular arrhythmias [3, 4]. Neuroglycopaenic symptoms, such as confusion, dizziness, weakness, reduced consciousness, unstable walking, lack of coordination and seizures are particularly serious in the elderly population due to the higher risk of fall-related bone fractures and injuries, together with the presence of other comorbidities, such as osteoporosis. Moreover, elderly patients are at higher risk of the hypoglycaemic episodes passing unnoticed by the patients themselves or even by the healthcare professionals that care for them as they can easily be confused with other diseases, such as transient ischaemic attacks or vasovagal episodes.

In any case, hypoglycaemia prevention requires different strategies, including regular education of the patient, as it has been shown that patient understanding of DM and its treatment decreases with time [5].

As regards frequency in the population, various studies have reported hypoglycaemia rates of between 0.02 and 0.35 episodes per type two diabetes patient per year [6]. However, the methodological differences between these studies make comparison of their results difficult. The main discrepancies result from the type of episodes observed (inclusion of episodes resolved by the patients themselves or only those requiring medical care or hospitalisation), choice of the population (patients with DM type 1 or 2, or both) or the definition of hypoglycaemia used (blood glucose levels established to consider an event as hypoglycaemic).

As regards the use of healthcare resources to treat hypoglycaemia in T2DM, the results vary according to studies undertaken in various countries. Estimation of these costs is affected by factors such as the incidence of hypoglycaemia, patient characteristics, an awareness of, and attitude towards, hypoglycaemic episodes by healthcare professionals and patients, variability in the quality of the care provided to these patients and the organisation of the healthcare systems [2]. A review of studies conducted in Spain estimated the cost of a single hypoglycaemic episode to be between €3000 and €3500 [7].

Very little has been published regarding the relationship between hypoglycaemic events and socioeconomic status. Leese *et al.* [8] observed an association between severe hypoglycaemia and increased socioeconomic deprivation. This was observed in patients with type 1 and 2 diabetes but was more strongly associated with type 1 diabetes.

In order to increase our understanding of this topic, this study, the aim of which was to determine the rate of patients who suffer severe hypoglycaemic episodes in the whole Basque Country population with T2DM, in other words those who require medical care in Accident and Emergency (A&E) departments or are admitted to hospital, according to their sociodemographic characteristics, and to estimate the annual healthcare costs for such patients, was designed.

## Methods

The study protocol was approved by the Ethical Committee of Clinical Research of Euskadi (PI2014074).

This is a descriptive study including all T2DM patients with public health insurance in the Basque Country in the period between 1 September 2010 and 31 August 2011. Osakidetza (Basque Public Health Service) has an electronic health record and other computerised sources in which information concerning all contact between its inhabitants and the public health service is recorded. In this system, diagnoses are coded according to the International Classification of Diseases (ICD-9-CM) [9], whereas the coding system used for medicinal products is the Anatomical, Therapeutic, Chemical classification system [10].

For this study, information concerning demographic and clinical variables was obtained from the PREST stratification database. This database combines information from various sources (primary and specialised care, A&E and hospital admittance registries) to obtain diagnoses, prescriptions and procedures. With this information, all citizens registered with Osakidetza are classified every year using the Adjusted Clinical Group (ACG) case-mix system [11]. A more detailed description of the database can be found in previous publications [12].

The study population was considered to be all T2DM patients residing in the Basque Country. To identify this population, all patients who, according to the PREST database, had received a diagnosis corresponding to T2DM or unspecified DM (including their complications) at any point in their life or who had been prescribed antidiabetic medication, irrespective of whether they had made use of care services during the observation period, were included. Those patients who had a diagnosis relating to type 1 DM at any contact or for whom all diagnoses corresponded to unspecified DM, but the only medication prescribed was insulin, were excluded from this group. As T2DM is rare in young people, an age limit of 34 years was established and only people above this threshold were analysed. As a result, a total of 134,413 people were considered to be T2DM patients.

Aggregated Diagnosis Groups (ADGs) were used to detect the presence of diseases other than diabetes in our study population. ADGs are a component of the ACG system and comprise 32 diagnostic categories, which are groups with similar severity, expected duration of the disease and treatment needs.

In addition, the glycosylated haemoglobin (HbA_1c_) values for the study patients were collected. In the case of patients with multiple determinations in the study period, only the last value recorded was considered.

The deprivation index from the census tract of residence was used as a socioeconomic variable [13]. This index is usually categorised into quintiles, although for this study the subjects were grouped into three categories, with category one corresponding to residents in the least deprived areas and category 3 those in the most deprived regions.

Health care provision were calculated by cost-weighted health care utilisation. We consider the cost of the following services: prescribing, primary care (including visits to physician and nurse, laboratory and radiology), specialised outpatient care (doctor visits, rehabilitation, dialysis, radiotherapy and chemotherapy services), A&E attendances and inpatients stays.

In the case of prescribing, the cost was computed directly from primary care prescriptions recorded in the electronic health records. For the other cases (visits to A&E, rehabilitation sessions, outpatient specialty care and primary care doctors and nurses; lab tests and X-rays requested in primary care; some procedures such as dialysis, radiation therapy or chemotherapy performed in day hospitals) the number of services used by each patient was multiplied by their standard cost. The costs of hospital stays and major outpatient surgical procedures were calculated according to the weights of their corresponding Diagnosis Related Groups (DRGs) [14].

Some services for which no information was available, namely admission to psychiatric hospitals, hospital-at-home and day hospital services (except the above-mentioned procedures), medical transport, prostheses and other equipment delivered to patients at home, were excluded from the cost estimate.

For study of severe hypoglycaemic episodes, we considered those which required hospital-based care (either resolved by A&E services or required hospitalisation). The population was classified into two groups: persons with no hypoglycaemic episode and those who presented one or more.

As the hypoglycaemic episodes recorded upon discharge from hospital are often incorrectly coded, a modification of the algorithm proposed by Ginde *et al.* was used [15]. These authors considered hypoglycaemic episodes to be:

1. Those episodes assigned the corresponding ICD-9-CM codes (251.00; 251.02; 251.10; 251.12; 251.20; 251.22).
2. Those related to intoxication with antidiabetic drugs (code 962.3).
3. Those in which the diagnosis was coded as 250.80 or 250.82 (diabetes with other specified complaints), excluding those which also presented one of the following diseases as co-diagnosis: cellulitis, lower limb ulcers, osteomyelitis, Oppenheim-Urban syndrome, diabetic lipidosis or secondary diabetic glycogenosis.

**Fig. 1:**
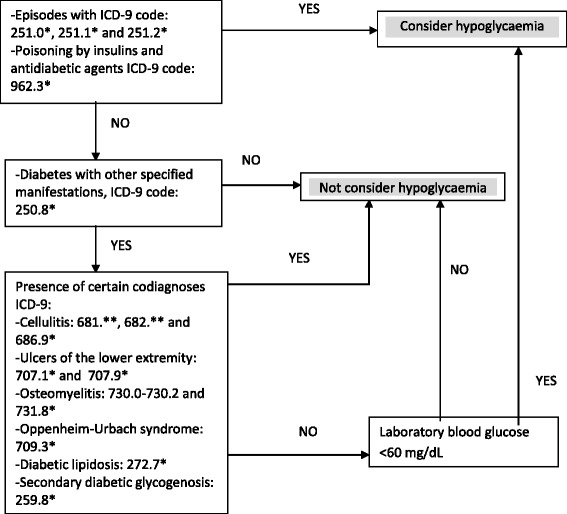
Method for the inclusion an episode of hypoglycaemia

Although the positive predictive value (PPV) for the episodes indicated by this third method in the study by Ginde *et al*. exceeded 79 % of correctly identified hypoglycaemic episodes, in our population the PPV did not reach 40 %. Consequently, in this latter case we decided to add an additional criterion, namely a laboratory analysis in which a blood glucose level of <60 mg/dL was recorded. Although this algorithm (Fig. 1) did not allow us to include hypoglycaemic episodes diagnosed using a reagent strip, it was found to be a specific method for detecting these episodes.

The mean was calculated for all continual variables and the frequency for discrete variables, stratifying by sex, age group and deprivation index. A logistic regression analysis was performed to study the effect of the independent variables (sex, age group, deprivation index, HbA_1c_ and ADG) on the probability of presenting a hypoglycaemic episode, and a linear regression analysis was performed to analyse the differences in annual healthcare costs per person. Values with *p* <0.05 were considered to be significant.

Statistical calculations were performed using Stata, Data Analysis and Statistical Software, Release 12 (StataCorp, LP, College Station, TX, USA).

## Results

Of the total population of 1,473,943 persons aged ≥35 years, 134,413 presented T2DM, which corresponds to a prevalence of 9.12 %. A total of 847 hypoglycaemic episodes were recorded in this group, therefore the average episodes/person/year was 0.63 %.

The total number of person with T2DM attended due to one or more hypoglycaemic episodes after one year was 747, which represents a percentage of 0.56 %. This value was higher in females (0.58 % vs. 0.53 % in males), in those subjects with a lower socioeconomic level (0.62 % for inhabitants of the most deprived regions) and in those belonging to the younger and older age groups. These values are summarised in Table 1.

**Table 1:** Stratification of patients who suffered from hypoglycaemic episodes in 2011 by age and deprivation index

	N people with T2DM	N (%) people with hypoglycaemic episodes	N males with T2DM	N (%) males with hypoglycaemic episodes	N females with T2DM	N (%) females with hypoglycaemic episodes
Distribution	134,413	747 (0.56)	72,537	386 (0.53)	61,876	361 (0.58)
Age range						
35-39	1,136	11 (0.97)	661	10 (1.51)	475	1 (0.21)
40-44	2,088	8 (0.38)	1,329	6 (0.45)	759	2 (0.26)
45-49	4,141	16 (0.39)	2,804	12 (0.43)	1,337	4 (0.30)
50-54	7,504	21 (0.28)	5,094	15 (0.29)	2,410	6 (0.25)
55-59	11,310	18 (0.16)	7,442	16 (0.21)	3,868	2 (0.05)
60-64	16,119	44 (0.27)	10,191	29 (0.28)	5,928	15 (0.25)
65-69	19,205	56 (0.29)	11,366	31 (0.27)	7,839	25 (0.32)
70-74	17,402	74 (0.43)	9,588	43 (0.45)	7,814	31 (0.40)
75-79	21,686	153 (0.71)	10,805	81 (0.75)	10,881	72 (0.66)
80-84	18,054	172 (0.95)	8,055	81 (1.01)	9,999	91 (0.91)
85+	15,768	174 (1.10)	5,202	62 (1.19)	10,566	112 (1.06)
Deprivation index						
1	20,917	113 (0.53)	11,926	61 (0.51)	8,991	52 (0.57)
2	53,801	266 (0.50)	29,659	145 (0.49)	24,142	121 (0.50)
3	59,695	368 (0.62)	30,952	180 (0.58)	28,743	188 (0.65)

N represents the sample size. Deprivation index 1 represents the residents in less deprived regions and 3 represents the residents in more deprived regions

However, the multivariate analysis showed no statistically significant differences between sexes or socioeconomic levels. Subjects with an HbA_1c_ level higher than 7 % and, in particular, younger subjects (aged 35–39 years) had a higher probability of presenting hypoglycaemia. The results of the logistic regression analysis are shown in Table 2.

**Table 2:** Odds ratio for the logistic regression model, with suffering a hypoglycaemic episode as dependent variable

People who have suffered at least one hypoglycaemic episode				
Characteristics	Odds ratio	*P* value	[95 %	Conf. interval]
Gender				
Males (reference)	1			
Females	1.140	0.194	0.935	1.390
Age ranges				
35-39 (reference)	1			
40-44	0.288	0.036	0.090	0.923
45-49	0.248	0.006	0.092	0.666
50-54	0.186	<0.001	0.074	0.466
55-59	0.123	<0.001	0.050	0.307
60-64	0.162	<0.001	0.070	0.376
65-69	0.153	<0.001	0.066	0.350
70-74	0.203	<0.001	0.090	0.458
75-79	0.274	0.001	0.124	0.607
80-84	0.336	0.007	0.151	0.745
85+	0.334	0.008	0.149	0.751
HbA_1c_				
HbA_1c_ ≤7 % (reference)	1			
HbA_1c_ >7 % and HbA_1c_ ≤ 8 %	1.651	<0.001	1.331	2.048
HbA_1c_ >8 % and HbA_1c_ ≤ 9 %	1.537	0.005	1.140	2.074
HbA_1c_ >9 %	2.216	<0.001	1.653	2.972
Deprivation index				
1 (reference)	1			
2	0.887	0.408	0.668	1.178
3	1.028	0.846	0.781	1.351

The ADGs are not provided in this table. Please contact the authors for further information

As regards costs, it was found that patients who presented hypoglycaemic episodes generated higher overall costs than those who did not suffer any such episode, with the overall difference being €7605 in males and €7280 in females (see Table 3). Thus, the primary and specialised outpatient care and prescription costs were twice as high in patients of both sexes with hypoglycaemic episodes, whereas Accident and Emergency and hospitalisation costs were five times as high.

**Table 3:** Average cost for a patient who has suffered one or more hypoglycaemic episode, by service and sex

Gender	Hypoglycaemic episode	Cost of primary care	Cost of specialised care	Cost of emergency care	Cost of hospitalisation	Cost of prescriptions	Total cost
Males	No	588	609	74	1442	760	3472
	Yes	1033	1248	426	6972	1397	11077
	Total	590	611	76	1467	762	3507
Females	No	660	563	80	1167	838	3307
	Yes	1142	1489	432	5979	1545	10587
	Total	662	567	82	1192	842	3345

A linear regression analysis was performed, adjusting the cost of the healthcare provided to T2DM patients for sociodemographics variables (age, sex and deprivation index), comorbidity (ADGs), HbA_1c_ levels and hypoglycaemic episodes (Table 4). This study showed that, on average, patients who presented a hypoglycaemic episode accounted for €2509 more of the overall costs (*p* <0.001). The average total cost was lower for females and increased with age up to 85 years. The differences based on glycaemic control level also reached statistical significance, with lower costs being observed for patients with HbA_1c_ levels <7 %, intermediate costs for those with a level between 7 % and 8 %, and the highest costs being found for patients with HbA_1c_ levels >8 %.

**Table 4:** Linear regression analysis with total cost as dependent variable

Total cost				
Characteristics	Coefficients	*P* value	[95 %	Conf. interval]
Gender				
Males (reference)	1			
Females	−129.3	<0.001	−183.6	−75.1
Age ranges				
35-39 (reference)	1			
40-44	25.8	0.893	−351.3	402.9
45-49	32.8	0.852	−311.5	377.1
50-54	79.3	0.636	−249.3	408.0
55-59	182.4	0.268	−140.1	504.9
60-64	340.9	0.036	22.1	659.6
65-69	363.4	0.025	46.0	680.9
70-74	412.0	0.011	93.7	730.4
75-79	481.5	0.003	164.0	799.0
80-84	422.6	0.01	103.2	742.0
85+	56.0	0.734	−267.4	379.4
One or more hypoglycaemic episode				
No (reference)	1			
Yes	2508.7	<0.001	2146.0	2871.3
Deprivation index				
1 (reference)	1			
2	59.6	0.135	−18.6	137.9
3	86.8	0.028	9.6	164.0
HbA_1c_				
HbA_1c_ ≤7 % (reference)	1			
HbA_1c_ >7 % and HbA_1c_ ≤ 8 %	251.5	<0.001	188.0	314.9
HbA_1c_ >8 % and HbA_1c_ ≤ 9 %	561.8	<0.001	467.1	656.6
HbA_1c_ >9 %	447.5	<0.001	340.9	554.0

The ADGs are not provided in this table. Please contact the authors for further information

## Discussion

Although methodological differences between studies make a comparison difficult, our findings are consistent with the literature as the percentage of people who suffered a hypoglycaemic episode (0.56 %) is in the range described by other authors [16–18]. Similarly, the results obtained as regards age ranges are also in accordance with those reported by other authors, with a higher proportion of episodes being observed in the younger age groups [19, 20].

Leese *et al.* [8] have reported that socioeconomic level is associated with the onset of a hypoglycaemic episode. Although it is true that the proportion of people who suffer at least one episode is higher in more deprived regions, the logistic regression analysis performed as part of our study did not find a statistically significant relationship between this factor and suffering at least one such episode or not.

As we have seen previously, there is a marked difference in the estimated direct cost per severe hypoglycaemic episode treated in hospital. Some authors have reported this quantity to be around €1370 in Spain [21], whereas others have reported values in the range €3000-3500 [7]. However, a comparison is impossible as the costs in our study are calculated on the basis of the total annual consumption of resources needed by a person who has suffered a hypoglycaemic episode rather than the cost of each hypoglycaemic episode.

In the Basque Country, the average difference in annual costs between people who presented one or more hypoglycaemic episode and those who did not was €7605 for males and €7280 for females. It should be noted, however, that only part of this cost is attributable to the event itself, with the remainder arising due to other characteristics of the patient, especially the co-existence of other health problems in that person. As such, after adjustment for these variables, the cost difference attributable to the presence of hypoglycaemic episodes decreased to €2509. The use of a sophisticated system for adjusting for comorbidity, namely the ACGs from Johns Hopkins University, the validity of which has been confirmed in several countries [22, 23], is one of the strengths of our study.

Another strength is the fact that, as the study is based on the entire T2DM population aged ≥35 years with public health insurance (in other words essentially the entire Basque Country population), selection biases are avoided. Moreover, a database containing information from primary care, in- and out-patient hospital care and pharmaceutical prescriptions is used for the inclusion criteria for T2DM patients. This is highly relevant as it has been reported that the use of a single source may result in inaccurate calculations [24, 25], whereas the combined use of various sources contributes to a better description of health problems [26].

One drawback of our study is that administrative databases only contain information regarding the problems for which people seek medical care. Moreover, it has proved difficult to identify hypoglycaemic episodes in our healthcare information systems as a marked under-reporting is commonly observed in medical notes and administrative databases. In addition, although laboratory results have also been used, diagnosis of a hypoglycaemic episode is often established using a reagent strip, for which no records are kept. Finally, some hypoglycaemic episodes are coded as “other complications” upon discharge from hospital. In light of the above, we have used a complex method which has allowed us to overcome some of these limitations.

## Conclusions

Hypoglycaemic episodes provoke various problems in clinical practice. Thus, symptoms (and the threshold for presenting them) vary and are often confused with other diseases or go unnoticed. In addition, the glucose levels accepted as pathological are not unique (various different standards have been proposed by various scientific societies [27]) and can often not be checked by the patient when suffering the episode. Their frequency is high, especially as type 2 diabetes progresses with time, and their onset cannot always be predicted. Moreover, compensation can often be inadequate or excessive.

As such, education, self-control and adequate treatment adherence are key objectives for managing this condition. Patients diagnosed with T2DM who have commenced pharmacological treatment must receive specific training in how to recognise and treat the first symptoms of hypoglycaemia, thereby reducing the possible complications arising from the episode. Moreover, the creation of a suitable database for measuring and monitoring these episodes would be advantageous as it could then be used to target interventions at specific sub-groups and to follow-up patients at high risk of hypoglycaemia due to their characteristics or previous hypoglycaemic episodes.

## Abbreviations

ACG: Adjusted clinical group
ADG: Aggregated diagnosis group
A&E: Accident and emergency
DM: Diabetes mellitus
HbA_1c_: Glycosylated haemoglobin
ICD-9-CM: International classification of diseases
PPV: Positive predictive value
PREST: Basque country stratification program
T2DM: Type 2 diabetes mellitus
